# Prognostic value of *PRR11* and immune cell infiltration in Ewing sarcoma

**DOI:** 10.1371/journal.pone.0299720

**Published:** 2024-03-01

**Authors:** Jian Wen, Lijia Wan, Xieping Dong

**Affiliations:** 1 Jiangxi Medical College, Nanchang University, Nanchang, Jiangxi, China; 2 Department of Orthopedics, JXHC Key Laboratory of Digital Orthopedics, Jiangxi Provincial People’s Hospital, The First Affiliated Hospital of Nanchang Medical College, Nanchang, Jiangxi, China; 3 Department of Child Healthcare, Hunan Provincial Maternal and Child Health Hospital, Changsha, Hunan, China; The University of Queensland Faculty of Medicine, AUSTRALIA

## Abstract

Ewing’s sarcoma (ES) is the second most common bone and soft tissue malignancy in children and adolescents with a poor prognosis. The identification of genes with prognostic value may contribute to the prediction and treatment of this disease. The GSE17679, GSE68776, GSE63155, and GSE63156 datasets were downloaded from the Gene Expression Omnibus database and qualified. Prognostic value of differentially expressed genes (DEGs) between the normal and tumor groups and immune cell infiltration were explored by several algorithms. A prognostic model was established and validated. Finally, functional analyses of the DEGs were performed. Proline rich 11 (PRR11) and mast cell infiltration were noted as the key indicators for the prognosis of ES. Kaplan–Meier and scatter plots for the training and two validation sets showed that patients in the low-*PRR11* expression group were associated with better outcomes than those in the high-*PRR11* expression group. The concordance indices and calibration analyses of the prognostic model indicated good predictive accuracy in the training and validation sets. The area under the curve values obtained through the receiver operating characteristic analysis for 1-, 3-, 5-year prediction were ≥ 0.75 in the three cohorts, suggesting satisfactory sensitivity and specificity of the model. Decision curve analyses suggested that patients could benefit more from the model than the other strategies. Functional analyses suggested that DEGs were mainly clustered in the cell cycle pathway. *PRR11* and mast cell infiltration are potential prognostic indicators in ES. *PRR11* possibly affects the prognosis of patients with ES through the cell cycle pathway.

## 1. Introduction

Ewing sarcoma (ES), characterized by the expression of a FET/ETS fusion oncoprotein, is the second most common primary bone malignancy in children, adolescents, and young adults [1, 2]. Thus far, the main treatment methods for ES are surgery, irradiation, and interval-compressed chemotherapy [3]. Moreover, with the development of new therapies for ES, the 5-year survival rate in patients with localized disease has increased to 70–80% [2]. However, significant improvement has not been achieved in the survival of patients with metastatic, recurrent, and refractory disease; the 5-year survival rate among such patients is 10–30% [2–4]. In addition, unfortunately, 20–25% of patients have metastases at initial diagnosis [5]. Therefore, ES remains an aggressive tumor associated with poor outcomes; it is imperative to identify new prognostic biomarkers and therapeutic targets, which may improve treatment success.

Due to the low incidence of ES (approximately 1.5 cases per million individuals) [2], it is difficult to conduct a large-sample study. Therefore, it may be useful to study ES based on data available in public databases. In this study, we downloaded data on ES from the Gene Expression Omnibus (GEO) database [6] and used bioinformatics methods to perform the analysis. Subsequently, the findings were validated in independent external cohorts, thereby confirming their robustness, reliability, and reproducibility.

Thus far, numerous new prognostic biomarkers and therapeutic targets have been discovered, such as *TrkC* [7], *STAG2* [8], *RRM2* [9], *miR-34a* [10], and *ATG2B* [11]. However, considering the complexity of ES and the major challenges in its treatment, further information on the prognosis of this disease is required. In addition, although cellular immunotherapy employing engineered T cells has demonstrated remarkable clinical efficacy in hematologic tumors, particularly B-cell malignancies [12]. However, in the case of ES, immunotherapy has displayed limited effectiveness despite the utilization of highly intensive therapies [13–15]. Thus, there is a critical need for further investigation into immune infiltration and the tumor microenvironment in ES. Therefore, this study aims to delve into the genes and immune-infiltrating cells that have the potential to influence the prognosis of ES.

## 2. Materials and methods

### 2.1 Data collection

The GSE17679, GSE68776, GSE63155, and GSE63156 datasets were downloaded from the GEO database (https://www.ncbi.nlm.nih.gov/geo/) on January 7, 2023, including expression, clinical, and survival data. The expression data were inspected to ensure that they were normalized, and the batch effect was removed using the “limma” package. An outlier was defined as a significantly different normalized expression profile of genes in a sample from that of the majority of samples in the group. Then, box plots and density plots were produced based on the expression data to perform quality control, and the outliers were removed [16].

### 2.2 Identification of differentially expressed genes (DEGs) between the healthy and tumor groups

The “limma” package was adopted to identify DEGs between the healthy and tumor groups in the GSE17679 and GSE68776 datasets, which included expression data of healthy subjects and patients with ES. Common DEGs with an absolute log_2_FoldChange >2 and adjusted *p*-value <0.01 (adjust method: “BH”) were selected for the next screening.

### 2.3 Identification of prognostic genes

Since the GSE17679 cohort was published earlier and the sample size was larger, we chose GSE17679 as the training set. Common DEGs were analyzed using univariate Cox regression analysis. Genes with a *p*-value <0.01 were included in the multivariate Cox regression analysis. Next, genes with a *p*-value < 0.01 were further subjected to least absolute shrinkage and selection operator (LASSO) regression analysis (λ = 1se) [17, 18]. The selected genes were evaluated using the Boruta feature selection, and the gene with the highest importance score was selected as an indicator for the prognostic model. Moreover, protein–protein interaction (PPI) network analysis of the proteins encoded by common DEGs was also performed to find hub proteins on the Search Tool for the Retrieval of Interacting Genes / Proteins (STRING) website (https://cn.string-db.org/) (interaction score ≥0.4) [19].

### 2.4 Assessment of immune cell infiltration and identification of tumor-infiltrating immune cells with prognostic value

Immune cell infiltration was estimated using the “xCell” package [20]. Thereafter, 64 types of infiltrating cells were assessed by univariate Cox regression analysis. Cells with a *p*-value <0.05 were included in the multivariate Cox regression analysis. Subsequently, tumor-infiltrating immune cells with a *p*-value <0.05 in the multivariate analysis were subjected to the Boruta feature selection, and the type of cells with the highest importance score was selected as another indicator for the prognostic model.

### 2.5 Construction and evaluation of a prognostic model in the training set

A prognostic model was established based on the identified hub gene and tumor-infiltrating immune cells. Kaplan–Meier (KM) analysis was used to preliminarily evaluate the prognostic value of the hub gene in the GSE17679 dataset. The characteristics of samples in the high- and low-expression groups of the hub gene were explored using the scatter plots. A nomogram was used to visualize the Cox proportional hazards regression model. Thereafter, the predictive accuracy and discriminatory capacity of the model were evaluated using the concordance index (C-index), calibration analysis, time-dependent receiver operating characteristic (ROC) analysis, and decision curve analysis (DCA) in the GSE17679 dataset.

### 2.6 Validation of the model in the GSE63155 and GSE63156 cohorts

KM analysis and scatter plots for the characteristics of samples were used to preliminarily evaluate the prognostic value of hub genes in the GSE63155 and GSE63156 cohorts. A nomogram was also used to visualize the Cox proportional hazards regression model in the GSE63155 and GSE63156 cohorts. Next, the predictive accuracy and discriminatory capacity of the model were also evaluated using the C-index, calibration analysis, time-dependent ROC analysis, and DCA in the GSE63155 and GSE63156 cohorts.

### 2.7 Functional analysis of DEGs between the healthy and tumor groups

Gene Ontology (GO) and Kyoto Encyclopedia of Genes and Genomes (KEGG) clustering [21], as well as gene set enrichment analysis (GSEA) [22], were performed to explore the functional enrichment of DEGs in the GSE17679 dataset. Hub gene co-expression DEGs were identified using the “cor.test” function (Pearson method) based on a *p*-value <0.05 and absolute correlation value ≥0.7. The PPI network analysis of the co-expression DEGs was performed on the STRING website (**https://cn.string-db.org/**) (interaction score ≥0.4).

### 2.8 Validation of proline rich 11 (PRR11) expression in tumor and normal bone tissues

We used immunohistochemistry to determine the expression of PRR11 in ES and normal bone tissues. Paraffin-embedded samples of 4 normal bone tissues and 4 osteosarcoma tissues were obtained from Jiangxi Provincial People’s Hospital (Nanchang, China). Primary and secondary antibodies were obtained from Bioss Inc. (Woburn, MA, USA; http://www.bioss.com.cn/, catalog number: bs-6237R) and Beijing Zhongshan Jinqiao Biotechnology Co., Ltd. (Beijing, China; http://www.zsbio.com/, catalog number: ZB-2301), respectively. All tissue sections were subjected to dewaxing, antigen retrieval by microwave in boiling antigen retrieval buffer (1mM EDTA, pH 8.0) for 20 minutes, elimination of endogenous peroxidase with 3% hydrogen peroxide for 10 minutes at room temperature, blocking with 5% bovine serum albumin V (Beijing Solarbio Science & Technology Co.,Ltd., Beijing, China; https://solarbio.com/gywm.php, catalog number: A8020) at 37°C for 30 minutes, incubation with primary antibody (1:400) overnight at 4°C, incubation with secondary antibody (1:100) for 30 minutes at room temperature, staining with 3,3’-diaminobenzidine for 3 minutes, restaining with hematoxylin, dehydration and sealing. The sections were examined using a ZEISS Axio Lab.A1 microscope (Carl Zeiss, Germany). For each sample slice, two randomly selected fields at 400× magnification were employed to calculate the positivity rate through ImageJ software. Following this, a comparison between the positivity rates of both groups was conducted and visualized using GraphPad Prism 8.3.0.

### 2.9 Ethics approval and consent to participate

This work was approved by the Ethics Committee of Jiangxi Provincial People’s Hospital (NO. 2022–059) and all experiments were performed in accordance with relevant guidelines and regulations. Informed consent was obtained from the participant.

### 2.10 Statistical analysis

In this study, the R software v3.63 was used to process data and generate charts. PPI network analyses were conducted on the STRING website (https://cn.string-db.org/) (interaction score ≥0.4) and visualized using the Cytoscape software v3.7.1 [23]. Cytohubba was used to identify the hub proteins in the Cytoscape software [24]. Flexible statistical methods were adopted for the statistical analysis; in the gene screening process, a more stringent significance threshold (0.01 rather than 0.05) was used to reduce the rate of false positives and obtain more reliable results. A flowchart outlining the study is available in S1 Fig.

## 3. Results

### 3.1 Data information and quality control

The GSE17679 dataset on the GEO website was submitted by the Laboratory of Cytomolecular Genetics, University of Helsinki (Helsinki, Finland) on August 17, 2009. This dataset included expression data of 64 ES tissue samples, 11 ES cell line samples, and 18 healthy subject samples, as well as clinical data of the 64 patients with ES. The expression data were detected by Affymetrix Human Genome U133 Plus 2.0 Array. The GSE68776 dataset on GEO website was submitted by University of Michigan (Ann Arbor, MI, USA) on May 11, 2015. This dataset included expression data of 32 ES biopsy specimens and 33 healthy subject tissue samples, which were detected by Affymetrix Human Exon 1.0 ST Array [transcript (gene) version]. The GSE63155 dataset on GEO website was submitted by the University of Michigan (Ann Arbor, MI, USA) on November 10, 2014. This dataset included expression and clinical data of 46 ES tissue samples, which were obtained from the Children’s Oncology Group Biorepository in Columbus (OH, USA). The expression data were detected by Affymetrix Human Exon 1.0 ST Array [transcript (gene) version]. The GSE63156 dataset on GEO website was submitted by the University of Michigan (Ann Arbor, MI, USA) on November 10, 2014. This dataset included expression and clinical data of 39 ES tissue samples, which were obtained from the EuroEwing tumor biorepository in Muenster, Germany. Expression data were detected by Affymetrix Human Exon 1.0 Array [transcript ST (gene) version].

**Fig 1:**
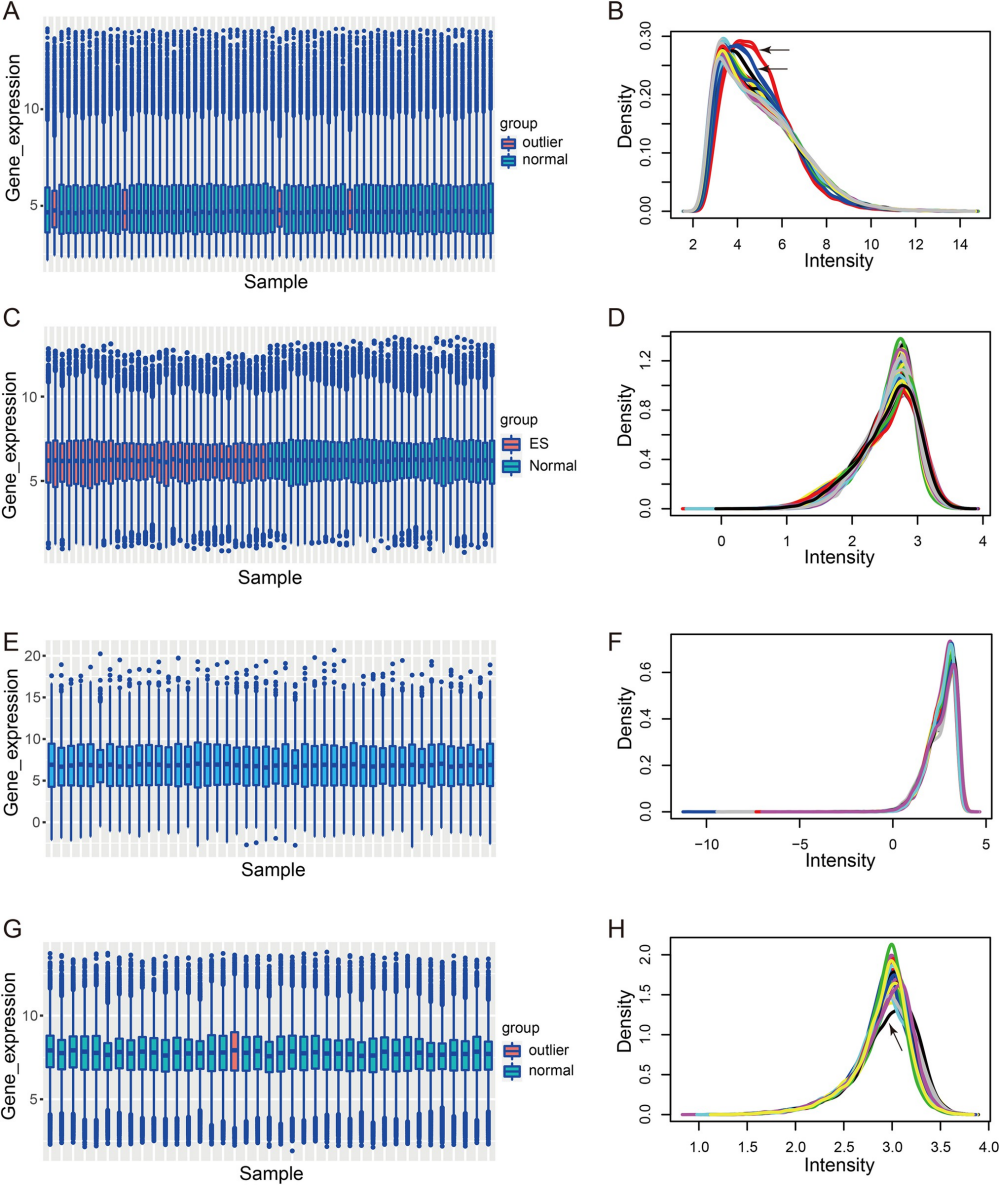
Quality control of the datasets. (**A**) Gene expression profile of the ES samples in GSE17679 by boxplot. (**B**) Gene expression profile of the ES samples in GSE17679 by density plot. (**C**) Gene expression profile of the samples in GSE68776 by the boxplot. (**D**) Gene expression profile of the samples in GSE68776 by density plot. (**E**) Gene expression profile of the samples in GSE63155 by boxplot. (**F**) Gene expression profile of the samples in GSE63155 by density plot. (**G**) Gene expression profile of the samples in GSE63156 by boxplot. (**H**) Gene expression profile of the samples in GSE63156 by density plot. ES, Ewing sarcoma.

Quality control of the data was performed by box plots and density plots. The median normalized gene expression values of GSM439887, GSM439897, GSM441000, and GSM441010 were higher than those of the other ES samples in GSE17679 (Fig 1A). Meanwhile, the distribution of the gene expression in these samples was significantly different from the other samples (Fig 1B). Therefore, they were defined as outliers and excluded from the subsequent prognostic analysis. In the normalized expression data of GSE63156, GSM1542396 was identified as an outlier (Fig 1G and 1H) and excluded from the subsequent prognostic analysis. The expression data of GSE68776 (Fig 1C and 1D) and GSE63155 (Fig 1E and 1F) were well normalized data without outliers and were utilized in the subsequent prognostic analyses.

### 3.2 Basic clinical characteristics of the training and validation sets

Basic clinical characteristics of the three sets are shown in S1 Table. The patient characteristics of the GSE63155 and GSE63156 datasets were comparable. Patients in the GSE17679 dataset were older than those in the GSE63155 and GSE63156 datasets. Sex distribution in these three datasets was similar.

### 3.3 DEGs between the healthy and tumor groups

**Fig 2:**
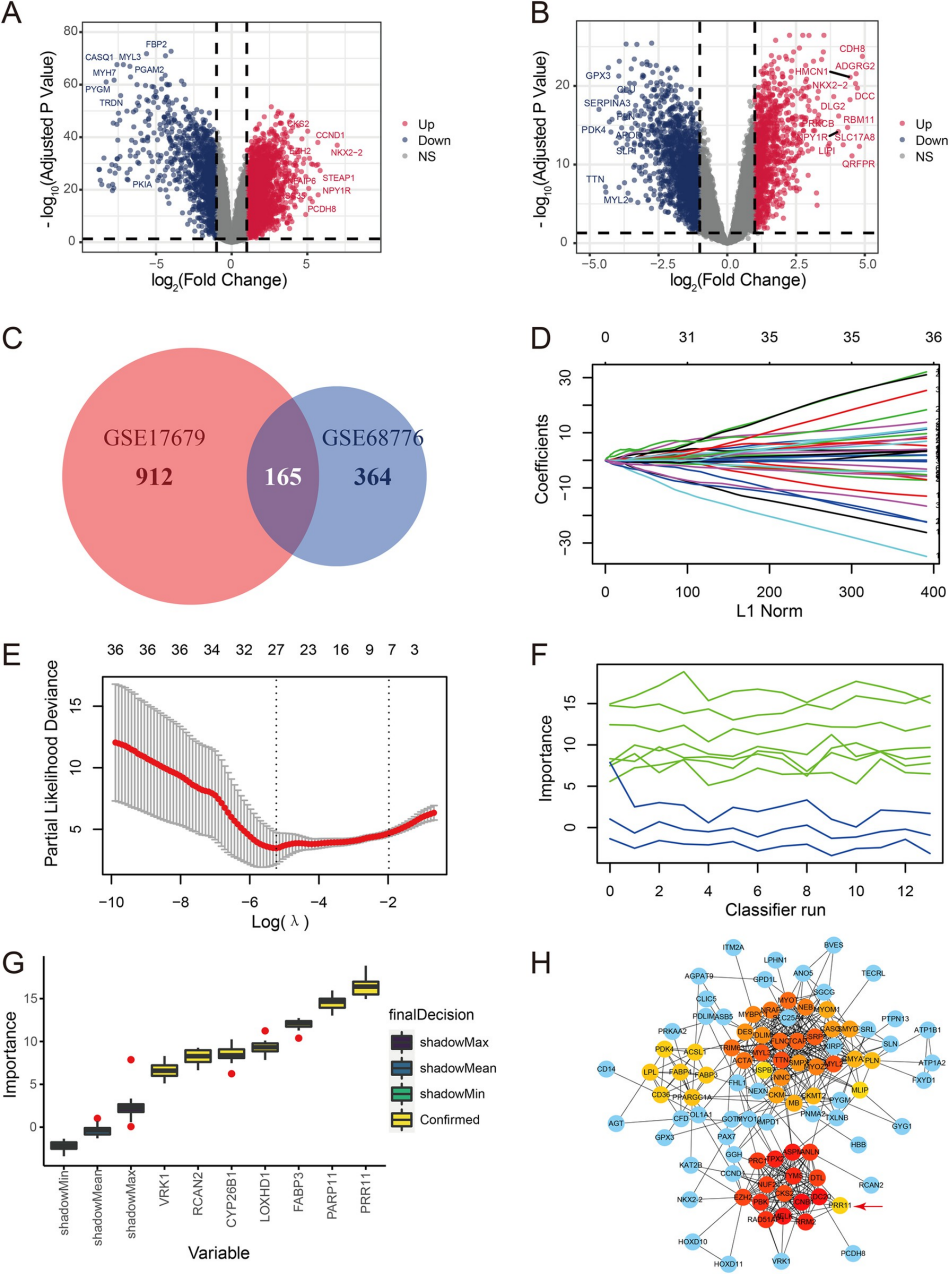
Identification of hub genes from common DEGs between healthy subjects and patients with ES. (**A**) Volcano plot of the DEGs (absolute log_2_FoldChange >1 and adjusted *p*-value <0.05) between healthy subjects and ES patients in GSE17679. (**B**) Volcano plot of the DEGs (absolute log_2_FoldChange >1 and adjusted *p*-value <0.05) between healthy subjects and patients with ES in GSE68776. (**C**) Venn plot for the DEGs (absolute log_2_FoldChange >2 and adjusted *p*-value <0.01) in GSE17679 and GSE68776. (**D**) LASSO coefficient profiles for the genes screened by univariate and multivariate Cox regression analyses in 10-fold cross-validations. (**E**) Partial likelihood deviance with alteration of the log(λ) plotted by LASSO regression in 10-fold cross-validations. (**F**) The importance score of the genes varied with the running times of the Boruta feature selection; the x-axis represents running times of the Boruta feature selection, and the y-axis represents the importance score. (**G**) Importance score of the genes screened by LASSO regression; the x-axis represents the genes screened by LASSO regression, and the y-axis represents the importance score calculated by the Boruta feature selection. (**H**) PPI network analysis for the common DEGs: red and yellow nodes indicate the top 50 genes by Cytohubba (color intensity indicates greater maximum clique centrality [MCC] value). DEGs, differentially expressed genes; ES, Ewing sarcoma; LASSO, least absolute shrinkage and selection operator; PPI, protein–protein interaction.

DEGs between the healthy and tumor groups were identified using the “limma” package with absolute log_2_FoldChange >1 and adjusted *p*-value <0.05 (Fig 2A and 2B). In addition, 1,077 DEGs in GSE17679 and 529 DEGs in GSE68776 were identified with absolute log_2_FoldChange > 2 and adjusted *p* value < 0.01. Then, 165 common DEGs were selected for the next screening (Fig 2C).

### 3.4 Identification of prognostic genes

The 165 common DEGs were evaluated by univariate and multivariate Cox regression analyses successively; 36 genes with a *p*-value <0.01 were retained. Subsequently, *PRR11*, *PARP11*, *FABP3*, *LOXHD1*, *CYP26B1*, *RCAN2*, and *VRK1* were analyzed by LASSO regression analysis (Fig 2D and 2E) and evaluated through the Boruta feature selection. *PRR11*, which showed the highest importance score in the Boruta feature selection, was selected as the hub gene for the prognostic model (Fig 2F and 2G). Subsequently, PPI network analysis of the proteins encoded by the 165 common DEGs was performed (Fig 2H). PRR11 was selected as a hub protein in the PPI network by Cytohubba (top 50 ranked by maximum clique centrality from high to low: CCNB1, CDC20, MELK, ASPM, TPX2, RRM2, TYMS, PBK, RAD51AP1, ANLN, PRC1, DTL, NUF2, EZH2, CKS2, TTN, MYL2, MYL3, CSRP3, TCAP, FLNC, MYOT, TRIM63, ACTA1, NRAP, NEB, MYOZ2, MYBPC1, TNNC1, PDLIM3, DES, SMPX, CASQ2, SMYD1, CKM, MYOM1, MB, CMYA5, CKMT2, PLN, PPARGC1A, FABP4, LPL, FABP3, ACSL1, CD36, PDK4, PRR11, MLIP, HSPB7).

### 3.5 Assessment of immune cell infiltration and identification of tumor-infiltrating immune cells with prognostic value

**Fig 3:**
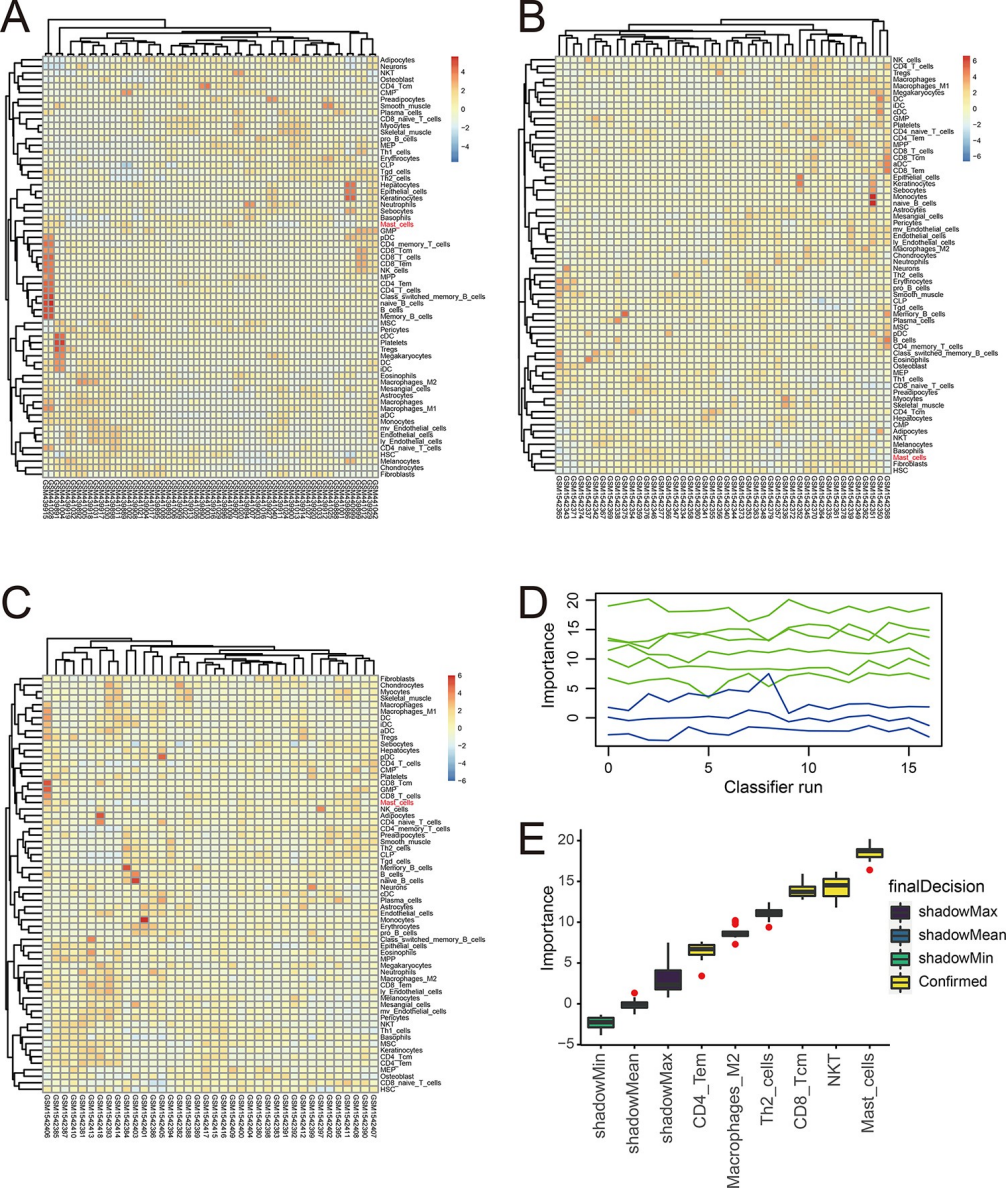
Cell infiltration profile in samples and tumor-infiltrating immune cells with prognostic value. (**A**) Cell infiltration profile of patients in GSE17679 by heatmap. (**B**) Cell infiltration profile of patients in GSE63155 by heatmap. (**C**) Cell infiltration profile of patients in GSE63156 by heatmap. (**D**) The importance score of the tumor-infiltrating immune cells varied with the running times of the Boruta feature selection; the x-axis represents the running times of the Boruta feature selection, and the y-axis represents the importance score of the variables. (**E**) Importance score of the tumor-infiltrating immune cells screened by the Boruta feature selection; the x-axis represents the tumor-infiltrating immune cells screened by the Boruta feature selection; the y-axis represents the importance score calculated using the Boruta feature selection. Th2 cells, T helper 2 cells; NKT, natural killer T cells; CD4 Tem, CD4^+^ T effector memory; CD8 Tcm, CD8^+^ T central memory cells.

A heatmap was used to display the data of the 64 tumor-infiltrating immune cells in samples in the training and validation sets (Fig 3A–3C). M2 macrophages, mast cells, T helper 2 cells, natural killer T cells, CD4^+^ T effector memory, and CD8^+^ T central memory cells, which had a *p*-value <0.05 both in the univariate and multivariate analyses (Table 1) were subjected to the Boruta feature selection (Fig 3D and 3E). Finally, mast cells were selected as the pivotal tumor-infiltrating immune cells for the prognosis of ES and another indicator for the prognostic model.

**Table 1:** Univariate and multivariate Cox analyses of tumor-infiltrating immune cells.

Immune infiltrating cells	Univariate Cox analysis			Multivariate Cox analysis		
	Hazard.Ratio	CI95	*P*.Value	Hazard.Ratio	CI95	*P*.Value
Macrophages_M2	1.82E+03	1.27E+01–2.60E+05	0.003	8.19E+10	1.78E+05–3.76E+16	**0**
Mast_cells	0.00E+00	0.00E+00–0.00E+00	0	0.00E+00	0.00E+00–0.00E+00	**0.001**
Th2_cells	9.12E+04	5.29E+01–1.57E+08	0.003	2.34E+13	1.91E+05–2.88E+21	**0.001**
NKT	5.74E+01	4.70E+00–6.99E+02	0.002	1.35E+05	2.00E+01–9.19E+08	**0.009**
CD4_Tem	0.00E+00	0.00E+00–3.10E-01	0.03	0.00E+00	0.00E+00–1.00E-02	**0.017**
CD8_Tcm	0.00E+00	0.00E+00–9.20E-01	0.047	0.00E+00	0.00E+00–1.20E-01	**0.023**
Skeletal_muscle	4.30E+02	1.13E+00–1.63E+05	0.045	0.00E+00	0.00E+00–2.01E+01	0.104
Erythrocytes	2.69E+10	4.73E+04–1.53E+16	0	0.00E+00	0.00E+00–4.12E+02	0.126
Eosinophils	7.35E+04	3.66E+02–1.48E+07	0	0.00E+00	0.00E+00–1.08E+02	0.165
Plasma_cells	2.62E+03	3.81E+00–1.81E+06	0.018	1.24E+02	0.00E+00–1.36E+08	0.497
CD4_T_cells	0.00E+00	0.00E+00–0.00E+00	0	0.00E+00	0.00E+00–1.03E+06	0.551
HSC	4.00E-02	0.00E+00–4.20E-01	0.007	2.10E-01	0.00E+00–4.37E+01	0.566
CD4_naive_T_cells	0.00E+00	0.00E+00–0.00E+00	0	7.52E+00	0.00E+00–4.15E+08	0.824

### 3.6 Construction and evaluation of a prognostic model in the training set

**Fig 4:**
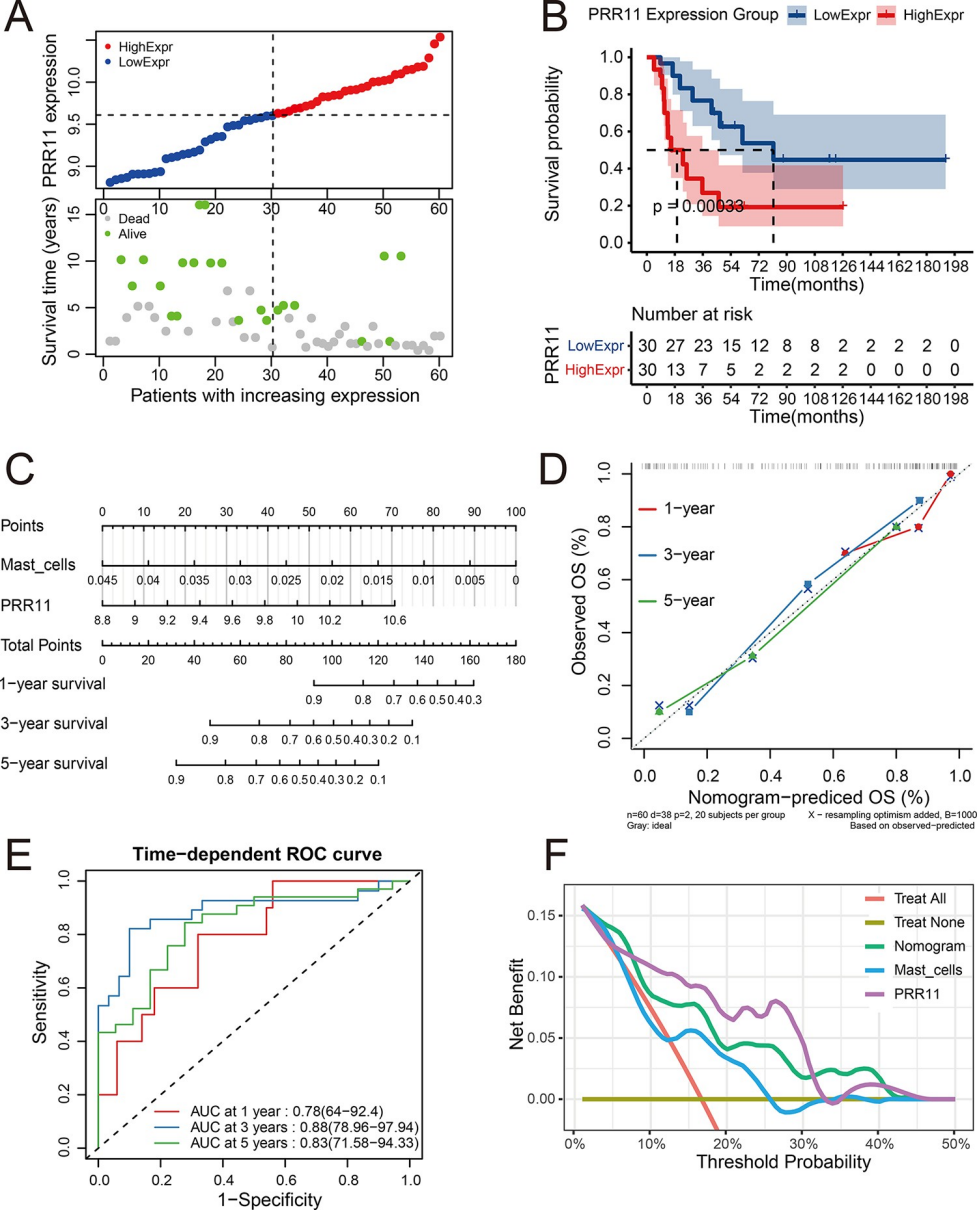
Evaluation of the prognostic value of *PRR11* and the prognostic model. (**A**) Scatter plots for the survival characteristics of patients with increasing *PRR11* expression in GSE17679; the left side of the vertical dashed line represents patients in the low-expression group, and the right side represents patients in the high-expression group. (**B**) KM plot for high- and low-*PRR11* expression groups in GSE17679. (**C**) Nomogram for the Cox proportional hazards regression model in GSE17679. (**D**) Calibration analysis of the model in GSE17679; the x-axis represents predicted overall survival rate by the model, and the y-axis represents observed overall survival rate; the diagonal (dashed line) refers to the ideal line. (**E**) Time-dependent ROC analysis of the model in GSE17679; the x-axis represents the 1-specificity of the model, and the y-axis represents the sensitivity of the model. (**F**) 1-year DCA in GSE17679; the x-axis represents the threshold probability for treatment or intervention, and the y-axis represents net benefit. DCA, decision curve analysis; KM, Kaplan–Meier; *PRR11*, proline rich 11; ROC, receiver operating characteristic.

The prognostic model established by *PRR11* and mast infiltrating cells in GSE17679 was as follows: Risk score = 1.376097 * *PRR11*–77.862471 * mast cells. A prognostic model was established using *PRR11* and mast cell infiltration. Characteristics of the samples in the high- and low-*PRR11* expression groups of the GSE17679 datasets are shown in Fig 4A. The upper part of the scatter plot shows the classification of patients into the high- and low-expression groups (median); the patients were ranked according to the expression of *PRR11*. The lower part of the scatter plot shows that patients in the high-expression group were associated with shorter overall survival and higher mortality rate than those in the low-expression group. The KM analysis showed that patients in the high-*PRR11* expression group had poorer outcomes than those in the low-expression group (*p* = 0.00033, log-rank test) (Fig 4B). The Cox proportional hazards regression model was visualized by a nomogram, which could predict the survival rate of patients by summing up the points of *PRR11* and mast cell infiltration according to their values (Fig 4C). The C-index of the model in GSE17679 was 0.776 (95% confidence interval [CI]: 0.735–0.817), suggesting a good predictive accuracy. Calibration analysis of the model showed that the broken lines were close to the ideal line (i.e., the predicted survival rate equaled the observed survival rate), which also suggested a good predictive accuracy for 1-, 3-, and 5-year outcomes (Fig 4D). Time-dependent ROC analysis showed good predictive sensitivity and specificity of the model; the values of the area under the curve (AUC) were 0.78, 0.88, and 0.83 for 1-, 3-, and 5-year prediction, respectively (Fig 4E). The DCA for 1-year prediction of the model and the *PRR11* and mast cell infiltration only strategies indicated that the model and the *PRR11* only strategies offered greater net benefit than the other strategies (Fig 4F).

### 3.7 Validation of the model

**Fig 5:**
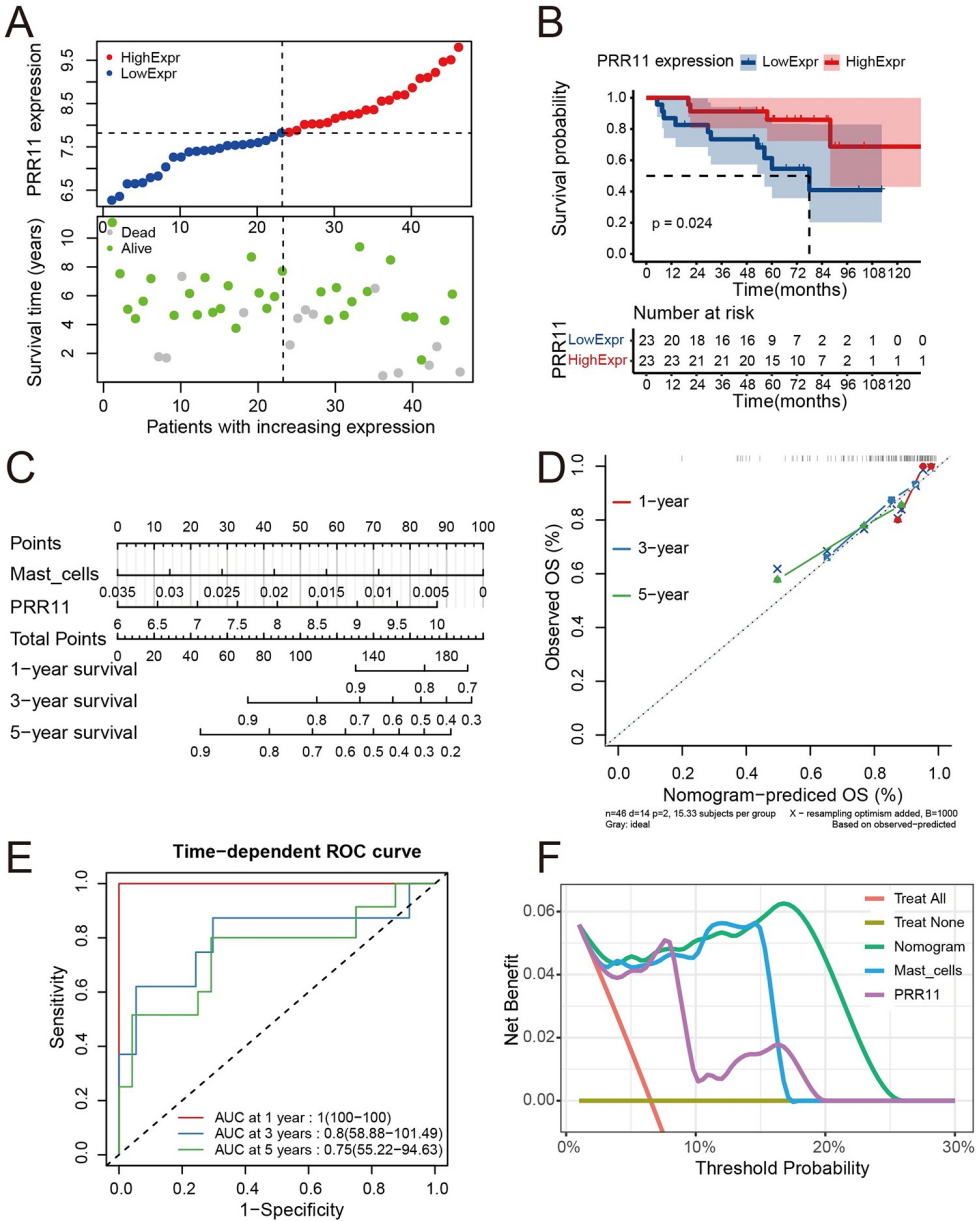
Validation of the model in the GSE63155 dataset. (**A**) Scatter plots for the survival characteristics of patients with increasing *PRR11* expression in GSE63155; the left side of the vertical dashed line represents patients in the low-expression group, and the right side represents patients in the high-expression group. (**B**) KM plot for the high- and low-*PRR11* expression groups in GSE63155. (**C**) Nomogram for the Cox proportional hazards regression model in GSE63155; the x-axis represents predicted overall survival rate by the model, and the y-axis represents observed overall survival rate; the diagonal (dashed line) refers to the ideal line. (**D**) Calibration analysis of the model in GSE63155. (**E**) Time-dependent ROC analysis of the model in GSE63155; the x-axis represents the 1-specificity of the model, and the y-axis represents the sensitivity of the model. (**F**) 1-year DCA in GSE63155; the x-axis represents the threshold probability for treatment or intervention, and the y-axis represents net benefit. DCA, decision curve analysis; KM, Kaplan–Meier; *PRR11*, proline rich 11; ROC, receiver operating characteristic.

Scatter plots for GSE63155 indicated that patients in the high-*PRR11* expression group were linked to shorter overall survival and higher mortality than those in the low-*PRR11* expression group (Fig 5A). The KM analysis showed that patients in the high-*PRR11* expression group had poorer outcomes than those in the low-*PRR11* expression group (*p* = 0.024, log-rank test) (Fig 5B). The nomogram of the Cox proportional hazards regression model (Risk score = 0.4348097 * *PRR11*–56.8561718 * mast cells) also demonstrated that *PRR11* was a risk factor, whereas mast cell infiltration was a protective factor for the prognosis of ES (Fig 5C). The C-index of the model in GSE17679 was 0.747 (95% CI: 0.665–0.829). Although this value was slightly lower than that obtained in the training set, it suggested a good predictive accuracy of the model. Calibration analysis of the model suggested a good predictive accuracy for 1-, 3-, and 5-year outcomes, with their corresponding broken lines approaching the ideal line (Fig 5D). Time-dependent ROC analysis showed good predictive sensitivity and specificity of the model; the AUC values were 1, 0.80, and 0.75 for 1-, 3-, 5-year prediction, respectively (Fig 5E). In the DCA for 1-year prediction, the prognostic model offered the highest net benefit versus other strategies (Fig 5F). Besides, the *PRR11* only and mast cell infiltration only strategies also offered greater net benefit than the treat-none and treat-all strategies (Fig 5F).

**Fig 6:**
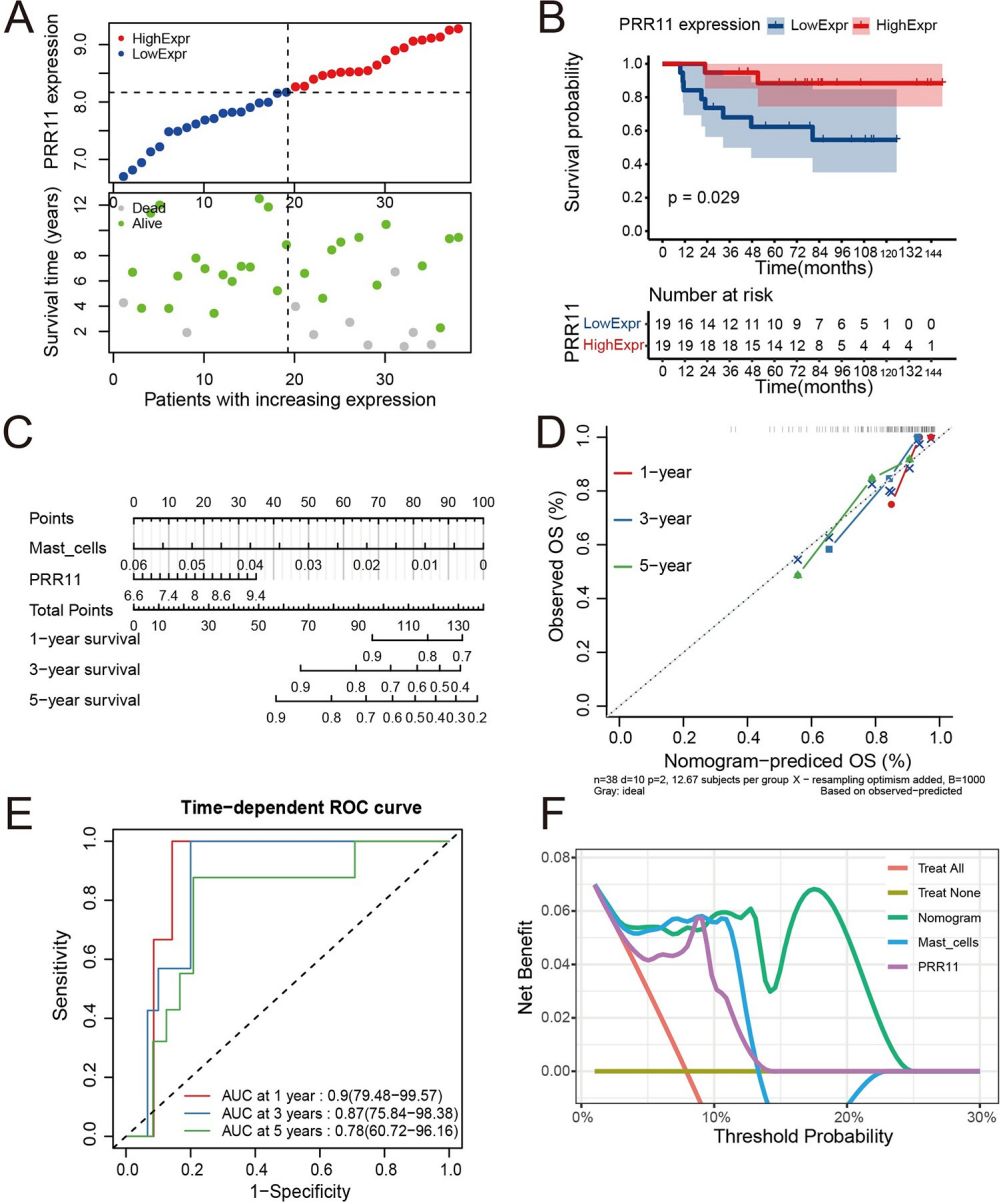
Validation of the model in the GSE63156 dataset. (**A**) Scatter plots for the survival characteristics of patients with increasing *PRR11* expression in GSE63156; the left side of the vertical dashed line represents patients in the low-expression group, and the right side represents patients in the high-expression group. (**B**) KM plot for the high- and low-*PRR11* expression groups in GSE63156. (**C**) Nomogram for the Cox proportional hazards regression model in GSE63156. (**D**) Calibration analysis of the model in GSE63156; the x-axis represents predicted overall survival rate by the model, and the y-axis represents observed overall survival rate; the diagonal (dashed line) refers to the ideal line. (**E**) Time-dependent ROC analysis of the model in GSE63155; the x-axis represents the 1-specificity of the model, and the y-axis represents the sensitivity of the model. (**F**) 1-year DCA in GSE63156; the x-axis represents the threshold probability for treatment or intervention, and the y-axis represents net benefit. DCA, decision curve analysis; KM, Kaplan–Meier; *PRR11*, proline rich 11; ROC, receiver operating characteristic.

Similar results were observed in the scatter plot of the GSE63156 dataset (Fig 6A). The KM analysis showed that patients in the high-*PRR11* expression group had poorer outcomes than those in the low-expression group (*p* = 0.029, log-rank test) (Fig 6B). The nomogram of the Cox proportional hazards regression model (Risk score = 0.4224908 * *PRR11*–56.3595245 * mast cells) also indicated that *PRR11* was a risk factor, whereas mast cell infiltration was a protective factor for the prognosis of ES (Fig 6C). The C-index of the model in GSE17679 was 0.786 (95% CI: 0.719–0.853). This value was higher than that recorded in the training set, thereby suggesting a higher predictive accuracy of the model. Calibration analysis of the model also suggested a good predictive accuracy for 1-, 3-, and 5-year outcomes, with their corresponding broken lines approaching the ideal line (Fig 6D). Time-dependent ROC analysis showed good predictive sensitivity and specificity of the model; the AUC values were 0.9, 0.87, and 0.78 for 1-, 3-, and 5-year prediction, respectively (Fig 6E). The DCA for 1-year prediction revealed that the prognostic model offered greater net benefit than other strategies (Fig 6F). Moreover, the *PRR11* only and mast cell infiltration only strategies also offered significantly greater net benefit than the treat-none and treat-all strategies (Fig 6F).

### 3.8 Functional analysis of DEGs between the healthy and tumor groups

**Fig 7:**
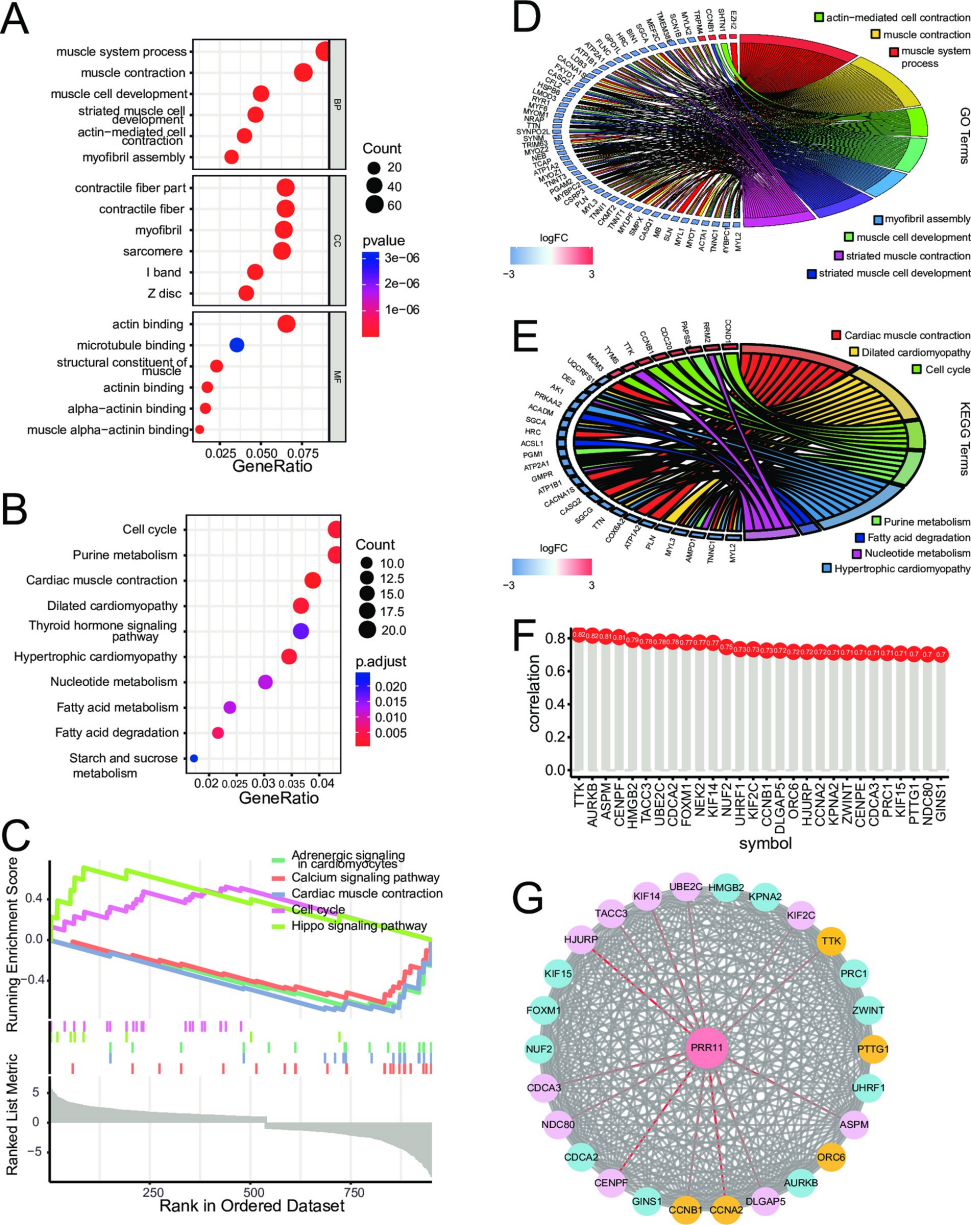
Differential analysis between the high- and low-risk score groups and functional analyses of DEGs. (**A**) Dot plot for GO clustering analysis of DEGs in GSE17679. (**B**) Dot plot for KEGG clustering analysis of DEGs in GSE17679. (**C**) GSEA analysis for DEGs in GSE17679. (**D**) Chord plot for the top seven clustered GO terms. (**E**) Chord plot for the top seven clustered KEGG pathways. (**F**) Lollipop plot for the common DEGs correlated with *PRR11* (absolute correlation value ≥0.7 and *p*-value <0.05). (**G**) PPI network analysis for proteins encoded by the top DEGs correlated with *PRR11* (*p*-value <0.05 and absolute correlation value ≥0.7). DEGs, differentially expressed genes; GSEA gene set enrichment analysis; GO, Gene Ontology; KEGG, Kyoto Encyclopedia of Genes and Genomes; PPI, protein–protein interaction; *PRR11*, proline rich 11.

The 1,077 DEGs in the GSE17679 dataset were clustered through GO and KEGG. The top three biological processes clustered by GO were muscle system process, muscle contraction, and muscle cell development; the top three cellular components were contractile fiber part, contractile fiber, and myofibril; the top three molecular functions were actin binding, microtubule binding, and structural constituent of muscle (Fig 7A and 7D). The top three clustered pathways by KEGG were cell cycle, purine metabolism, and cardiac muscle contraction (Fig 7B and 7E). The top gene sets clustered by GSEA were cell cycle, Hippo signaling pathway, adrenergic signaling in cardiomyocytes, calcium signaling pathway, and cardiac muscle contraction (Fig 7C). The cell cycle and Hippo signaling pathway were upregulated in patients with ES, whereas the other three gene sets were downregulated. The correlation analysis demonstrated that *PRC1*, *AURKB*, *UHRF1*, *CCNA2*, *UBE2C*, *CENPF*, *GINS1*, *FOXM1*, *HJURP*, *ZWINT*, *ORC6*, *ASPM*, *TACC3*, *NUF2*, *KIF15*, *CCNB1*, *KIF14*, *DLGAP5*, *CDCA2*, *NDC80*, *HMGB2*, *CDCA3*, *KIF2C*, *PTTG1*, *CENPE*, *NEK2*, *TTK*, and *KPNA2* were DEGs co-expressed with *PRR11* (absolute correlation value ≥0.7 and *p*-value <0.05) (Fig 7F). The PPI network analysis of proteins encoded by the co-expressed genes revealed potential direct or indirect interactions with PRR11 (Fig 7G). Among the proteins encoded by the 28 co-expressed genes, CCNAB2, CCNAB1, ORC6, PTTG1, and TTK were involved in the cell cycle pathway (yellow nodes in Fig 7G).

### 3.9 PRR11 expression in ES and normal bone tissues

**Fig 8:**
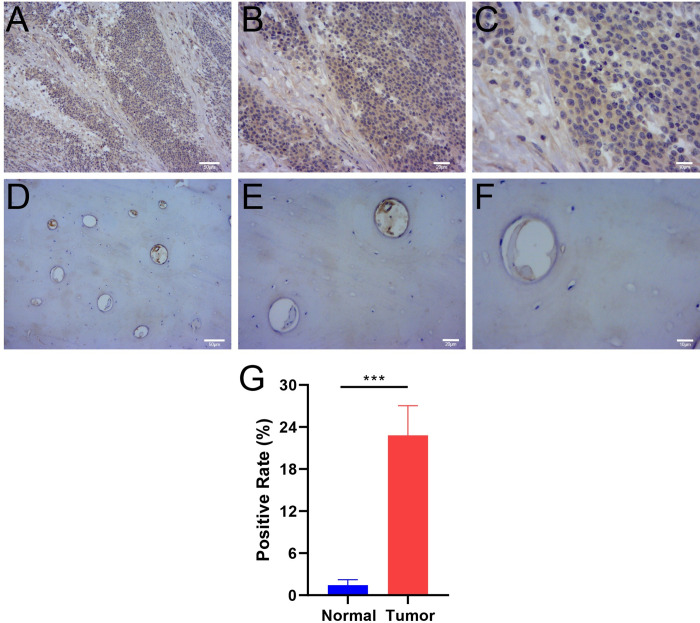
Expression of PRR11 in ES and normal bone tissues. Immunohistochemical staining of PRR11 in ES tissue. Magnification: ×100 (A), ×200 (B) and ×400 (C): PRR11 was stained brown in the cytoplasm of ES cells. Immunohistochemical staining of PRR11 in the normal bone tissue. Magnification: ×100 (D), ×200 (E) and ×400 (F): there was no significant brown staining of areas in the normal bone tissue. (G) The barplot illustrates the positive rate of PRR11 in immunohistochemical staining, analyzed using the Mann-Whitney test (non-normal distribution), with the presentation of Mean ± Standard Error of the Mean. Significance level: no significance (ns), p ≥ 0.05; *, p < 0.05; **, p < 0.01; ***, p < 0.001, ****, p < 0.0001. ES, Ewing sarcoma; PRR11, proline rich 11.

In the ES tissue, the cytoplasm of ES cells was stained brown (Fig 8A–8C). In contrast, in the normal bone tissue (cortical bone tissue), the extracellular matrix and cells in bone were not stained (Fig 8D–8F). The expression of PRR11 in ES tissues was notably elevated compared to that in normal tissues (*p* = 0.0002) (Fig 8G).

## 4 Discussion

In this study, rigorous quality control of the data was conducted to ensure intra- and inter-group comparability, as well as the reliability of conclusions. Batch effects were removed within the datasets and outliers were excluded by box plots and density plots (Fig 1A–1H). The common DEGs between healthy subjects and patients with ES in the GSE17679 and GSE68776 datasets were selected for further analysis (Fig 2A–2C). Based on the Cox regression analyses, LASSO regression analysis, and the Boruta feature selection, *PRR11* was identified as the hub gene for the prognosis of ES (Fig 2D–2G). The results of the PPI network analysis also suggested the importance of PRR11 (Fig 2H). Besides, the results of the immunohistochemical staining confirmed the high expression of PRR11 in ES tissues. Thereafter, immune cell infiltration scores were calculated using the “xCell” package, and mast cell infiltration was selected as another indicator for the prognostic model (Fig 3A–3E).

The prognostic value of *PRR11* was evaluated through KM analysis and scatter plots in the GSE17679 dataset. The results demonstrated that high expression of *PRR11* was associated with poor prognosis of ES (Fig 4A and 4B). This was also confirmed by the results of the nomogram. Moreover, the nomogram in GSE17679 also indicated that mast cell infiltration was a protective factor for the prognosis of ES (Fig 4C). The predictive accuracy of the model was evaluated using the C-index and calibration analysis. The results (Fig 4D) indicated good predictive accuracy in the training set. Time-dependent ROC analysis also suggested good predictive sensitivity and specificity of the model (Fig 4E). The DCA confirmed the prognostic value of *PRR11* and mast cell infiltration in ES (Fig 4F). In addition, when the threshold was set <30%, the *PRR11* only strategy offered a greater net benefit than the model. In GSE63155 and GSE63156, the results of the KM (*p* < 0.05) and scatter plots analyses were similar to those recorded in the GSE17679 dataset; high expression of *PRR11* was associated with poor prognosis of ES (Figs 5A and 5B and 6A and 6B). The results of the C-index and calibration analyses (Figs 5C and 6C) of the model also indicated a good predictive accuracy in GSE63155 and GSE63156. The values of the C-index were similar in the three datasets. Time-dependent ROC analyses in GSE63155 and GSE63166 suggested good predictive sensitivity and specificity of the model (highest and lowest AUC values: 1 and 0.75, respectively) (Figs 5E and 6E). Particularly, the AUC values for 1- and 3- year prediction were ≥0.8. The results of the DCA of the model in the GSE63155 and GSE63156 datasets were similar. At most thresholds, the model offered greater net benefit than the other strategies (Figs 5F and 6F).

Functional analysis of the DEGs between healthy subjects and patients with ES suggested that they were associated with contractile fiber contraction and the cell cycle process (Fig 7A and 7B). The GSEA indicated that the cell cycle pathway was likely to be upregulated in patients with ES (Fig 7C). Our results suggested that the cell cycle was likely to be activated in patients with ES and closely associated with the proliferation of ES cells [25, 26]. Core clustered genes in different processes are shown in Fig 7D and 7E. *CCND1*, *CDC20*, *CCNB1*, *TTK*, and *MCM3* were the top clustered genes in the cell cycle process. The co-expression of *PRR11* and DEGs was also examined (Fig 7F). *TTK*, *AURKB*, *ASPM*, and *CENPF* were the most correlated DEGs.

*TTK* encodes a protein which is essential for chromosome alignment at the centromere during mitosis, as well as for centrosome duplication. It is a critical mitotic checkpoint protein for accurate segregation of chromosomes by serine/threonine kinases; it participates in the regulation of alignment and segregation of chromosomes during mitosis and meiosis through association with microtubules [27–29]. *ASPM* encodes a protein which is essential for normal mitotic spindle function in embryonic neuroblasts and might play a role in mitotic spindle regulation [30–32]. *CENPF* encodes a protein which is a component of the nuclear matrix during the G2 phase of the interphase, and may play a role in chromosome segregation during mitosis [33, 34]. PPI network analysis of the co-expressed DEGs also suggested interaction between these proteins. KIF2C, CCNA2, UBE2C, CENPF, HJURP, ASPM, TACC3, CCNB1, KIF14, DLGAP5, NDC80, CDCA3, and TTK might directly interact with PRR11. These are mainly involved in mitosis and the cell cycle, especially the segregation of chromosomes during mitosis. In summary, *PRR11* possibly influences the prognosis of ES mainly by affecting mitosis and the cell cycle. High expression of *PRR11* may promote the proliferation of ES cells, thus leading to poor prognosis of patients.

*PRR11* is a protein-coding gene located in the cytoplasm and nucleus; it is involved in the regulation of the cell cycle (provided by Alliance of Genome Resources, April 2022). Zhang et al. found that *PRR11* could regulate progression from the late-S to the G2/M phase and induce premature chromatin condensation [35]. Another study revealed that *PRR11* could promote cell proliferation by regulating PTTG1 through interaction with the transcription factor E2F1 in the pan-cancer setting [36]. In non-small cell lung carcinoma, *PRR11* drives F-actin assembly by recruiting the actin-related protein 2/3 complex [37]. In the present study, the main clustered cellular components identified by GO analysis was contractile fiber. Moreover, the main clustered molecular functions were actin and microtubule binding, which affect the segregation of chromosomes during mitosis. This is consistent with the previously reported role of *PRR11* in non-small cell lung carcinoma [35, 37]. Furthermore, *PRR11* has been associated with other types of lung cancer [38], clear cell renal cell carcinoma [39], tongue squamous cell carcinoma [40], pancreatic cancer [41], gastric cancer [42], osteosarcoma [43], breast cancer [44], etc. It possibly exerts effects on the cell cycle and autophagy, eventually leading to tumorigenesis, progression, and poor prognosis of patients. To the best of our knowledge, this study is the first to demonstrate that *PRR11* could also act as a prognostic biomarker for ES.

In the immune microenvironment of Ewing’s sarcoma, the downregulation of Human Leukocyte Antigen (HLA) A, B, and C on the surface of tumor cells hampers the recognition of tumor-associated antigens by antigen-presenting cells and effector T cells [13]. Concurrently, elevated levels of HLA-G and an increase in regulatory T cells (Tregs) further impede the activation of tumor-specific T cells. In addition to bolstering the presence and activity of Tregs, F2 fibrocytes and myeloid-derived suppressor cells can also secrete cytokines that suppress cytotoxic T cell responses [13]. Some tumor-infiltrating immune cells, such as CD3 ^+^ and CD8^+^ tumor-infiltrating lymphocytes, have been reported to be associated with a favorable prognosis for ES [14, 45]. Nevertheless, histologically, limited immune cell infiltration has been reported in ES [2, 13, 46]. In our study, we found that low mast cell infiltration was associated with poor prognosis in patients with ES. In a study by de Silva MV et al., involving a comparison of 26 cases of common synovial sarcoma and poorly differentiated synovial sarcoma, it was observed that samples with mast cell infiltration were more likely to be associated with common synovial sarcoma rather than poorly differentiated synovial sarcoma [47]. Ren et al. also reported analogous discoveries through the application of machine learning techniques [48]. However, the role of mast cell infiltration in tumors was less well-defined [49–52]. It has been shown that mast cells are involved in the regulation of various physiological functions (e.g., vasodilation, angiogenesis, bacterial, and parasite elimination) and could influence the infiltration of other immune cells (e.g., dendritic cells, tumor-associated macrophages, and lymphocytes) [51]. Therefore, mast cells might influence the prognosis of ES through their inherent functions. Moreover, mast cell infiltration might also be involved in tumor osteolysis, as they were almost exclusively present at the tumor-bone interface, which might be associated with tumor invasion and metastasis [53]. Furthermore, it’s well-established that the proto-oncogene c-Kit was highly expressed on the surface of ES cells [54–57]. Hence, we speculated that the highly expressed c-Kit on the surface of ES cells, could competitively bind stem cell factors with stem cell factor receptors on the surface of mast cells, thereby impeding mast cell proliferation. However, further investigation is necessary to determine the mechanism through which mast cells affect the prognosis of patients with ES.

In summary, we found that *PRR11* and mast cell infiltration have prognostic value in ES. Thereafter, a prognostic model was established and successfully validated using two independent external cohorts from different geographical locations (i.e., USA and Europe). The predictive accuracy and discriminatory capacity of the model was satisfactory in the training and validation sets. According to the present results, *PRR11* was likely to promote the cell cycle of tumor cells and might be associated with the prognosis of patients with ES.

The limitations of this study should be acknowledged. Firstly, information from the public databases is limited. Many indicators of potential interest are not included in the primary data. Secondly, due to the low incidence of ES, it is difficult to conduct studies with a large sample size. Therefore, additional data will contribute to enhancing the reliability and stability of the current findings.

## 5 Conclusions

*PRR11* and mast cell infiltration are potential prognostic indicators in ES. *PRR11* possibly affects the prognosis of patients with ES through the cell cycle pathway.

## Supporting information

S1 Fig The flow chart of this study. (DOCX)

S1 Table Clinicopathological characteristics in the training and validation sets. (DOCX)
