# Supplementary figures and images for: Prognostic value of PRR11 and immune cell infiltration in Ewing sarcoma
Source: PLoS One. 2024 Mar 1;19(3):e0299720. doi: 10.1371/journal.pone.0299720 (PMC10906862; doi:10.1371/journal.pone.0299720)

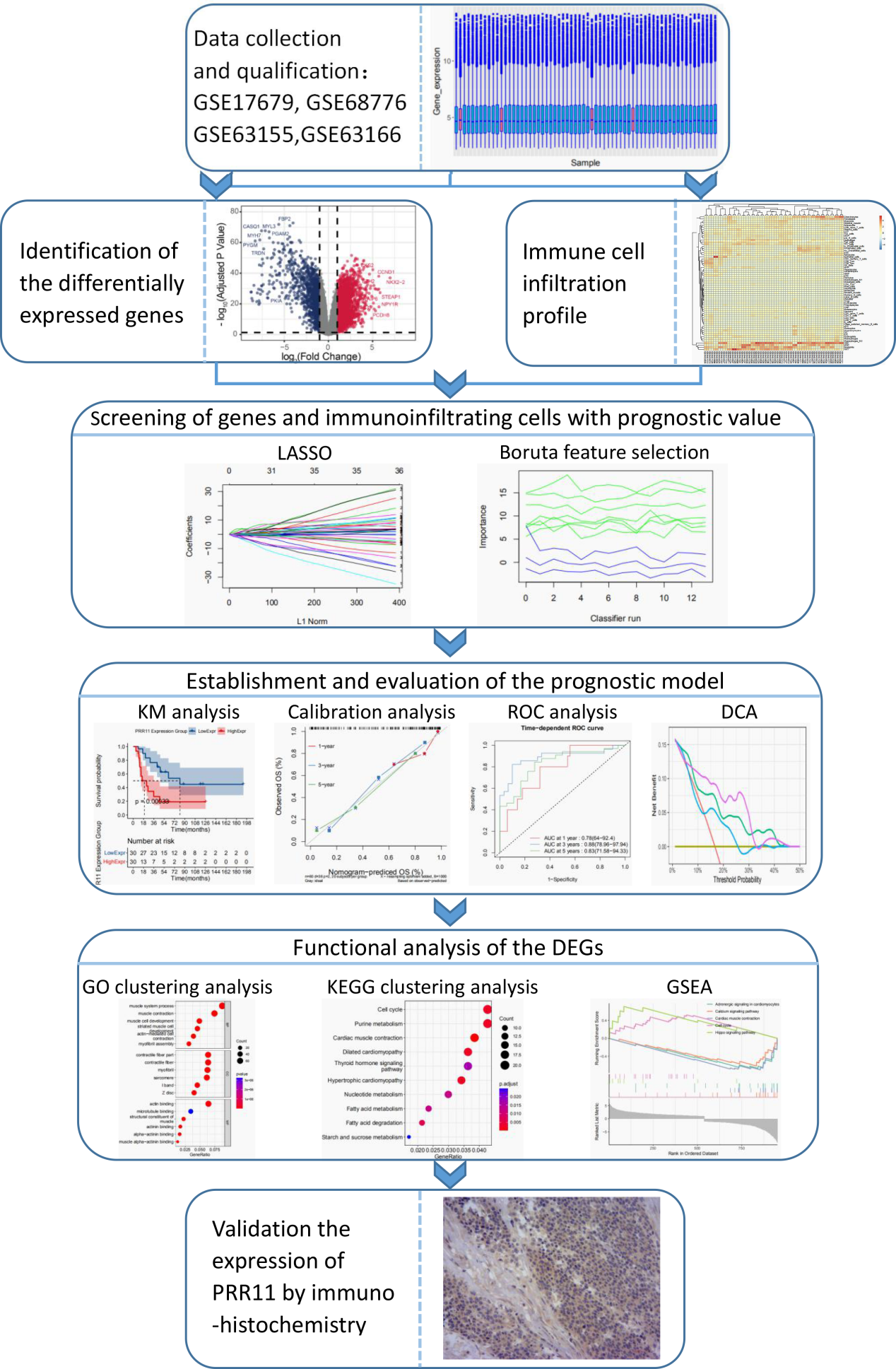


**S1 Figure** The flow chart of this study.

Supplement: S1 Fig — (DOCX) [file pone.0299720.s001.docx]
