# Supplementary material for: Prognostic value of PRR11 and immune cell infiltration in Ewing sarcoma
Source: PLoS One. 2024 Mar 1;19(3):e0299720. doi: 10.1371/journal.pone.0299720 (PMC10906862; doi:10.1371/journal.pone.0299720)
Supplement: S1 Table — (DOCX) [file pone.0299720.s002.docx]

**S1 Table Clinicopathological characteristics in the training and validation sets**

| Charateristics | Levels | GSE17679 | GSE63155 | GSE63156 |
| --- | --- | --- | --- | --- |
| Size |  | 60 | 46 | 39 |
| Age (years:median  [Q1,Q3]) |  | 18.00  [15.00, 23.00] | 13.12 ^a***^  [9.39, 15.50] | 14.00 ^a***^  [7.90, 17.12] |
| Sex | Female | 20 (33.3%) | 19 (41.3%) | 20 (51.3%) |
|  | Male | 40 (66.7%) | 27 (58.7%) | 19 (48.7%) |
| OS.time (median [Q1,Q3]) |  | 38.65  [15.60, 62.60] | 60.32 ^a*^  [52.04, 77.29] | 79.00 ^a**^  [45.77, 107.45] |
| OS | Alive | 22 (36.7%) | 32 (69.6%) ^b**^ | 28 (71.8%) ^b**^ |
|  | Dead | 38 (63.3%) | 14 (30.4%) ^b**^ | 11 (28.2%) ^b**^ |

GSE17679 was used as a control group. ^a^ refers to Kruskal-Wallis Rank Sum Test, ^b^ refers to Fisher’s Exact Test, * p < 0.05, ** p < 0.01, *** p < 0.001.
